# Differences in Distribution and Biological Effects of F_3_O_4_@PEG Nanoparticles in Normotensive and Hypertensive Rats—Focus on Vascular Function and Liver

**DOI:** 10.3390/biomedicines9121855

**Published:** 2021-12-07

**Authors:** Andrea Micurova, Michal Kluknavsky, Silvia Liskova, Peter Balis, Martin Skratek, Ludmila Okruhlicova, Jan Manka, Iveta Bernatova

**Affiliations:** 1 Centre of Experimental Medicine, Institute of Normal and Pathological Physiology, Slovak Academy of Sciences, 813 71 Bratislava, Slovakia; Andrea.Micurova@savba.sk (A.M.); Michal.Kluknavsky@savba.sk (M.K.); Silvia.Liskova@gmail.com (S.L.); Peter.Balis@savba.sk (P.B.); 2 Institute of Pharmacology and Clinical Pharmacology, Faculty of Medicine, Comenius University, 811 08 Bratislava, Slovakia; 3 Institute of Measurement Science, Slovak Academy of Sciences, 841 04 Bratislava, Slovakia; Martin.Skratek@savba.sk (M.S.); Jan.Manka@savba.sk (J.M.); 4 Centre of Experimental Medicine, Institute of Heart Research, Slovak Academy of Sciences, 841 04 Bratislava, Slovakia; Ludmila.Okruhlicova@savba.sk

**Keywords:** iron oxide nanoparticles, blood pressure, nitric oxide, liver, arteries, gene expression, iron metabolism, nuclear factors

## Abstract

We investigate the distribution and biological effects of polyethylene glycol (PEG)-coated magnetite (Fe_3_O_4_@PEG) nanoparticles (~30 nm core size, ~51 nm hydrodynamic size, 2 mg Fe/kg/day, intravenously, for two days) in the aorta and liver of Wistar–Kyoto (WKY) and spontaneously hypertensive rats (SHR). Fe_3_O_4_@PEG had no effect on open-field behaviour but reduced the blood pressure (BP) of Fe_3_O_4_@PEG-treated SHR (SHRu) significantly, compared to both Fe_3_O_4_@PEG-treated WKY (WKYu) and saline-treated control SHR (SHRc). The Fe_3_O_4_@PEG content was significantly elevated in the aorta and liver of SHRu vs. WKYu. Nitric oxide synthase (NOS) activity was unaltered in the aorta, but significantly increased in the liver of SHRu vs. SHRc. In the aorta, Fe_3_O_4_@PEG treatment increased *eNOS, iNOS, NRF2*, and *DMT1* gene expression (considered main effects). In the liver, Fe_3_O_4_@PEG significantly elevated *eNOS* and *iNOS* gene expression in SHRu vs. SHRc, as well as *DMT1* and *FTH1* gene expression (considered main effects). Noradrenaline-induced contractions of the femoral arteries were elevated, while endothelium-dependent contractions were reduced in SHRu vs. SHRc. No differences were found in these parameters in WKY rats. In conclusion, the results indicated that the altered haemodynamics in SHR affect the tissue distribution and selected biological effects of Fe_3_O_4_@PEG in the vasculature and liver, suggesting that caution should be taken when using iron oxide nanoparticles in hypertensive subjects.

## 1. Introduction

Metal nanoparticles (NPs), including ultra-small superparamagnetic iron oxide nanoparticles (USPIONs), possess strong potential for various biomedical applications [1,2,3]. It is well-known that the properties and biological effects of USPIONs are considerably dependent on their size, physicochemical factors, and surface modification.

Amongst metal NPs, magnetite (Fe_3_O_4_) is used mainly due to its higher magnetization [4], while neutral biocompatible polyethylene glycol (PEG) is used as it has been approved by the Food and Drug Administration [5]. PEGylation has been shown to reduce the toxic effects of uncoated iron oxide NPs [6,7], including oxidative damage to DNA, proteins, and membrane lipids, as well as interference with the innate iron metabolism in mammals [2].

The potential negative effects of iron oxide nanoparticles (IONs) in vivo result from various factors [8]—mainly from their degradation followed by free iron release after decomposition of their coating. Elevated free iron levels may lead to oxidative stress, as well as affecting biogenic iron homeostasis, through altered production of hepatic hormone hepcidin (encoded by *HAMP* gene), a main regulator of iron absorption in the enterocyte [9,10]. In addition, degradation of USPIONs can alter ferritin (FTH1) expression, which is a main iron-storage protein in the liver, as well as the expression of iron cell transporters such as divalent metal transporter 1 (DMT1) and transferrin receptor 1 (TfR1). Iron-induced oxidative stress can further activate the expression and/or activity of antioxidant defence system enzymes; mainly superoxide dismutase (SOD). In addition, USPIONs may alter the expression of the nuclear factor erythroid 2-related factor 2 (NRF2), a nuclear transcription factor involved in cytoprotection in low-grade oxidative stress, inflammation, and metabolic alterations [11,12,13,14,15]. Moreover, several studies have confirmed the reciprocal regulation of NRF2 and peroxisome proliferator-activated receptor gamma (PPARγ) [16]. PPARγ is involved in the regulation of energetic metabolism, lipogenic pathways, and anti-inflammatory mechanisms. Increased PPARγ expression is also characteristic of liver damage [17,18].

In our previous study [19], we showed that a single acute i.v. administration of PEG-coated USPIONs had no effect on BP and heart rate, serotonin-induced contractions, and overall endothelium-dependent relaxation in normotensive WKY rats, when determined 100 min post-administration. In addition, we found that USPIONs elevated nitric oxide (NO) production and the release of superoxide in the liver and aorta. The highest levels of USPIONs (determined by biomagnetometry) were found in the blood and aorta in WKY, while lower levels were positively determined in the liver, kidneys, and left heart ventricles [20]. It was of interest that exposure of USPION-treated rats to acute stress, associated with a sudden BP increase, led to a decreased USPION-derived iron content in blood [19]. In addition, iron oxide nanoparticles can potentially alter the blood–brain barrier and enter the brain, suggesting their possible neurotoxic and behavioural effects through modulation of neurotransmitter release. Among others, NO is a well-known neurotransmitter and neuromodulator affecting BP regulation, as well as endothelium-derived relaxing factor. A lack of NO—notably in the vascular bed—led to reduced vasorelaxation and elevated vasoconstriction, followed by hypertension development [21,22]. On the other hand, elevated production of NO—notably in the liver—by inducible nitric oxide synthase (iNOS) and/or endothelial nitric oxide synthase (eNOS), which has been associated with liver dysfunction, can lead to reduced systemic vascular resistance and hypotension. Thus, normal liver function is necessary for normal vascular and cardiac functions [23].

The above-mentioned findings inspired us to perform a study in which we tested the hypothesis that chronically elevated BP (hypertension) may affect the tissue distribution and selected physiological, metabolic, and genomic effects of USPIONs, manifested by changes in spontaneous behaviour, as well as by alterations in vascular and/or liver functions, which may be associated with changes in the expression of several genes involved in regulating NO production, redox state, and iron metabolism in the arteries (e.g., aorta or femoral arteries) and liver.

Thus, the aim of this study was to evaluate selected biological (i.e., behavioural, metabolic, and genomic) effects of Fe_3_O_4_@PEG nanoparticles in conditions of chronic high blood pressure, compared to normotension. We investigated whether high BP affects the presence of USPIONs in the bloodstream and their incorporation into selected tissues, which could be associated with increased USPION-derived iron content in the aorta and liver, and manifested by altered spontaneous behaviour, BP changes, altered vascular function, and genomic changes. Collectively, the main purpose of this study was to evaluate whether chronically high BP is a factor that should be taken into account when USPIONs are used for diagnostic or therapeutic purposes in subjects with hypertension.

## 2. Materials and Methods

### 2.1. Experimental Design

In this study, we used normotensive WKY (*n* = 14) and spontaneously hypertensive (*n* = 16) male rats at the age of 14–16 weeks. All rats were born in the certified animal facility of the Institute of Normal and Pathological Physiology, Centre of Experimental Medicine, Slovak Academy of Sciences, in order to maintain a standardized environmental background for all animals. Rats were fed with pelleted chow for young rats deficient in phytoestrogens (Altromin 1314, variant P; Altromin Spezialfutter, Lage, Germany) until their ninth week of age. Afterward, rats were fed with Altromin 1324 chow (for adult rats). The iron content in both chows was 192.51 mg/kg. The pellet food and water were available ad libitum. Rats were housed under standard conditions at 22–24 °C, humidity 45–65%, and with a 12 h light/dark cycle.

Before the experiment, rats had a catheter implanted in the jugular vein, for infusion of USPIONs or saline (in the controls). The catheters were implanted under 2.5–3.5% isoflurane anaesthesia. The procedure for catheter implantation has been described in detail by Liskova et al. [19]. Rats were randomly divided into control WKY rats (WKYc), USPION-treated WKY rats (WKYu), control SHR rats (SHRc), and USPION-treated SHR rats (SHRu), with *n* = 7–8 per group. During the 10 min infusion, rats were placed in a plastic box with dark walls and a transparent lid (27 cm × 14 cm × 9 cm in size), which allowed for their free movement. USPIONs were administered intravenously on two consecutive days, at a dose of 2 mg Fe per kg of body weight per day. Rats were killed by decapitation after brief exposure to CO_2_ (until the loss of consciousness) approximately 26 h after the second infusion, and trunk blood, tissues, urine, and faeces were collected for further analyses. Faeces were collected randomly from those excreted during the open-field test, keeping approximately the same time of excretion after USPION infusion. Serum was collected from trunk blood. Urine was collected from the bladder after decapitation. The design of the experiment is provided in Table 1.

We used commercially available PEG-coated magnetite USPIONs. The USPIONs were purchased from Sigma-Aldrich (Bratislava, Slovakia, cat. No. 747408, PubChem SID 329765832, accessed on 18 March 2021). The iron content was 1 mg Fe/mL, and the concentration of NPs dispersed in water was 0.034 nmol/mL. The size of the USPIONs core (determined by transmission electron microscopy) was 28–32 nm, the zeta potential was –12 mV, the polydispersity index was 0.1, and the hydrodynamic size was about 45 nm (parameters declared by the manufacturer). USPIONs were autoclaved at 121 °C for 30 min. Before their use, the exact physico-chemical properties of the USPIONs were further determined, as published in detail in our earlier publication [20]. USPIONs were then dispersed with sterile saline to reach a final dose of 2 mg of Fe/kg of body weight in a final volume of 1 mL and infused intravenously into the jugular vein during the 10 min infusion using an infusion pump. Sterile saline (1 mL) was infused into control rats.

All of the chemicals used in this study were purchased from Sigma–Aldrich (Bratislava, Slovakia) and Merck Chemicals (Bratislava, Slovakia), unless stated otherwise.

### 2.2. Open-Field Test

Locomotor activity and anxiety-like behaviour were measured using an open-field test (OF) between 07:00–07:30 h, using the Any-maze (Stoelting Europe, Dublin, Ireland) video tracking system in 10 min trials. The testing conditions have been previously described in detail by Kluknavsky et al. [24]. The total distance travelled in the OF and immobility time were determined as the parameters of locomotor activity. As the markers of anxiety-like behaviour, the relative distance travelled in the central zone (calculated as the percentage of distance travelled in the central zone with respect to total distance travelled), time spent in the central zone (central time) and counts of entries into the central zone (central entries) were determined.

### 2.3. Systolic Blood Pressure and Heart Rate

The systolic BP and HR were measured in pre-conditioned conscious rats using the non-invasive tail-cuff plethysmography method, as previously described by Puzserova et al. [25]. Each value was calculated as the average of five measurements. The BP and HR values were measured at the beginning of the experiment (basal) and 24 h after the second infusion (end).

### 2.4. Nitric Oxide Synthase Activity

NOS activity was determined on the basis of conversion of [^3^H]-L-arginine (specific activity 5 GBq/mmol, ~100,000 dpm; ARC, St. Louis, MO, USA) to [^3^H]-L-citrulline, using 20% tissue homogenates (*w*:*v*) of the brainstem, aorta, and liver through the method described in detail previously [19]. The enzyme activity was expressed as pkat/g of protein. The protein concentration was determined using the Lowry method.

### 2.5. Gene Expression

The mRNA expression levels of the *eNOS, iNOS, NRF2, PPARγ*, antioxidant enzymes superoxide dismutase 1 and 2 (*SOD1* and *SOD2*), *DMT1, TfR1*, and *β-actin* (a housekeeping gene) were determined using a real-time quantitative polymerase chain reaction (RT-qPCR).

The total RNA of the samples was isolated by using PureZOL™ RNA Isolation Reagent (Bio-Rad, Hercules, CA, USA), according to the manufacturer’s protocols. The amount of total isolated RNA was spectrophotometrically quantified at 260/280 nm and 260/230 nm, using a NanoDrop spectrophotometer (Thermo Scientific, Waltham, MA, USA). In the next step, the isolated RNA was reverse transcribed into cDNA using Eppendorf Mastercycler (Eppendorf AG, Elbmarsch, Germany) and the iScript™ cDNA Synthesis Kit (Bio-Rad, Hercules, CA, USA) reaction mixture, according to the manufacturer’s instructions. Gene amplification was performed using qPCR on a CFX96 Real-Time PCR detection system (Bio–Rad, Hercules, CA, USA). SsoAdvanced Universal SYBR Green Supermix (Bio-Rad, Hercules, CA, USA) was used for gene amplification. Primer pairs used to amplify selected genes are listed in the Table 2.

### 2.6. Vascular Functions

**Figure 1:**
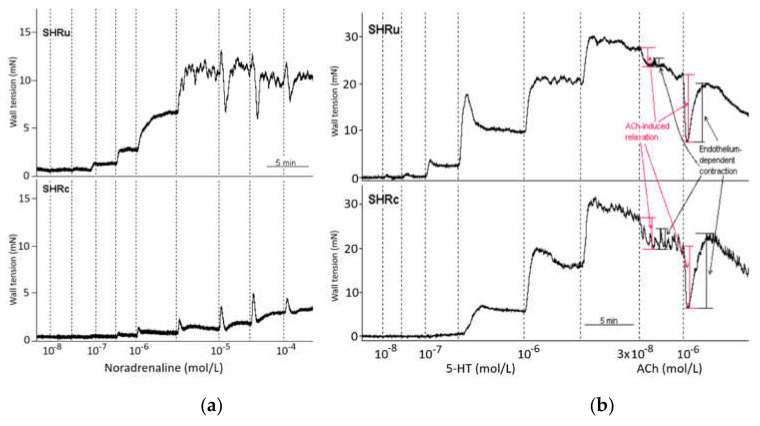
An example of the original recording of noradrenaline—(**a**) and serotonin-induced (**b**) contractions and acetylcholine-induced endothelium-dependent relaxations, followed by spontaneous endothelium-dependent contractions in the femoral arteries of control and USPION-treated rats. Abbreviations: ACh, acetylcholine; SHRc, control spontaneously hypertensive rats; SHRu, USPION-treated spontaneously hypertensive rats; NA, noradrenaline; 5-HT, 5-hydroxytryptamine (serotonin).

Isolated and cleaned fresh femoral arteries, with intact endothelium, were cut into segments and placed in a Mulvany–Halpern isometric myograph (Dual Wire Myograph system 410A, Danish Myo Technology A/S, Aarhus, Denmark). The myograph chambers were filled with modified physiological salt Krebs–Henseleit solution (PSS; containing in mmol/L: 119 NaCl, 4.7 KCl, 1.17 MgSO_4_.7H_2_O, 25 NaHCO_3_, 1.18 KH_2_PO_4_, 0.03 Na_2_EDTA, 2.5 CaCl_2_.2H_2_O, 5.5 glucose; 37 °C, pH 7.4) and bubbled with 95% O_2_ and 5% CO_2_. The inner arterial diameter of the femoral arteries was set to 90% of the diameter predicted for the pressure at 100 mmHg in the wire myograph. After 30 min of stabilisation, the arteries achieved their basal tones. To test the viability of isolated arteries, the arteries were incubated in a depolarising solution; that is, modified Krebs–Henseleit solution in which NaCl was exchanged for an equimolar (125 mmol/L K^+^; KPSS) concentration of KCl for 2 min. After being washed out, the experimental protocol was carried out. After the washing and stabilisation of the basal tone, cumulative concentrations of 5-hydroxytryptamine (5-HT, 10^−8^–10^−5^ mol/L) or noradrenaline (NA, 10^−8^–10^−4^ mol/L) were used to determine concentration-response curves. This was followed by application of acetylcholine (ACh; 3 × 10^−8^ and 10^−6^ mol/L), in order to induce endothelium-dependent relaxations. After maximal ACh-induced relaxation was achieved, the endothelium-dependent contraction developed within the next 3 min, as shown in Figure 1a,b. EC_50_, E_max_, and slope were calculated using Hill’s equation, and endothelium-dependent ACh-induced relaxations were calculated as the % of maximal 5-HT contraction. Endothelium-dependent ACh-induced contractions were calculated as the % of maximal ACh-induced relaxation for each ACh concentration used (3 × 10^−8^ and 10^−6^ mol/L).

### 2.7. Determination of the USPIONs Content

Determination of USPION-originated iron content in the serum, urine, faeces, aorta, and liver was performed by measuring their magnetic properties, as described previously by Skratek et al. [20]. USPION content in blood was calculated as a sum of serum content plus USPION content found previously in erythrocytes [26]. Briefly, a Quantum Design (San Diego, CA, USA) SQUID magnetometer MPMS-XL 7AC was used. Magnetic characterization of USPIONs, saline-treated control (both SHRc and WKYc), and Fe_3_O_4_@PEG-treated (SHRu and WKYu) was carried out by measuring the field dependence of the mass magnetization (*M*) at a temperature of 300 K, using an applied magnetic field up to 1 T. The USPION-originated iron content in the USPION-treated rats was calculated as *M*’ = *M*_SAMPLEu_ − *M*_c_ (where *M*_SAMPLEu_ is magnetization of the tissue sample of USPION-treated rat, and *M*_c_ is the average magnetization of the given tissue from control rats) and determined by comparison to *M*_USPION_, which is magnetization of USPIONs with a declared concentration of iron [20]. This method distinguishes USPION-originated iron from iron naturally present in the tissues, on the basis of their different magnetic properties under the given experimental conditions. Tissues for magnetic measurements were dissected using ceramic scissors and forceps, cleaned of connective tissues, and immediately frozen in liquid nitrogen. All samples were kept at −80 °C until magnetometric measurements were performed.

### 2.8. Transmission Electron Microscopy of the Tissues

Aortae of WKYu and SHRu rats were cleaned of connective tissue (*n* = 3 for each group) and cut into 3 mm long rings. Liver tissue was cut into small pieces, about 2 mm^3^ in size. Tissues were fixed by immersion in 2.5% glutaraldehyde in 0.1 mol/L cacodylate buffer (Serva, Heidelberg, Germany) at a pH of 7.4, for 3 h at 40 °C. After washing in the buffer, the samples were post-fixed in 1% OsO_4_ for 30 min, dehydrated in an alcohol series, infiltrated in propylene oxide, and embedded in Epon 812 (Serva, Heidelberg, Germany). Ultrathin sections of the aortae were cut using a Leica ultramicrotome (Leica Microsysteme EM UC7, Wien, Austria), and then mounted on nickel grids [27]. Unstained sections were examined using Tesla BS 500 transmission electron microscope (Brno, Czech Republic), in order to detect the distribution of nanoparticles. Semi-quantitative evaluation was used to determine the differences in amount and distribution of USPIONs in the aorta and liver of WKYu and SHRu. Due to unaltered endothelium-dependent relaxation and increased NA-induced contractions, we focused on the presence of USPIONs in the tunica media of the aorta. For both tissues investigated, the score was defined from minus (−), indicating the absence of USPIONs, to (+++), as their highest amount.

### 2.9. Statistical Analysis

Statistical analyses of blood pressure (absolute values), heart rate (absolute values), and open-field behaviour were performed using three-way ANOVA for repeated measures, with measurement (basal and end) as a repeated factor. Treatment (saline in controls or USPIONs) and rat strain (WKY and SHR) were the independent factors. Two-way ANOVA (strain and treatment) was used for all other analyses (except for magnetometric measurements). All ANOVA analyses were followed with Bonferroni’s post hoc test. Normality of the data distribution was tested using the Kolmogorov–Smirnov test. Magnetometric measurements were analysed by two-tailed Student’s *t*-test. Correlations between variables were analysed using Pearson’s correlation coefficient (*r*). The values were considered to differ significantly when *p* < 0.05. The results are presented as mean ± standard error of means (SEM). The GraphPad Prism v7.02 software (GraphPad Software, Inc., San Diego, CA, USA) and Statistica v13.5 (StatSoft Europe, Hamburg, Germany) was used for the statistical analyses.

## 3. Results

### 3.1. Open-Field Behaviour

**Figure 2:**
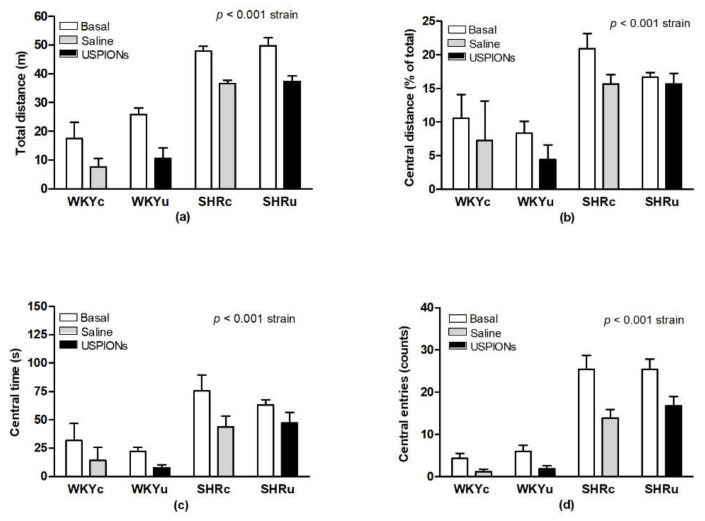
Total distance travelled (**a**), relative distance travelled in the central zone (**b**), time spent in the central zone (**c**), and central entries in the open-field test (**d**). Results are mean ± SEM. See the Results section for detailed 3-way ANOVA of results. Abbreviations: USPIONs, ultra-small superparamagnetic iron oxide nanoparticles; WKYc, control Wistar–Kyoto rats; WKYu, USPION-treated Wistar–Kyoto rats; SHRc, control spontaneously hypertensive rats; SHRu, USPION-treated spontaneously hypertensive rats.

ANOVA revealed significant strain-dependent differences for total distance travelled (Figure 2a) in the open-field (F(1,23) = 151.45, *p* < 0.0001), with significantly higher locomotor activity in SHR compared to WKY (48.82 ± 1.58 m vs. 22.00 ± 2.97 m). Repeated testing (i.e., measurement) significantly reduced the total distance travelled of all rats (F(1,23) = 42.64, *p* < 0.001). The main effects of treatment, as well as the interactions of the factors, were statistically insignificant. In agreement with these findings, immobility of SHR rats was significantly reduced compared to WKY (187.82 ± 9.78 s vs. 378.82 ± 19.20 s; F(1,23) = 155.48, *p* < 0.0001). Distance travelled in the central zone, calculated as the percentage of the total distance travelled in the OF was significantly higher in SHR, compared to WKY (18.82 ± 1.58 m vs. 9.39 ± 1.58 m; F(1,23) = 17.17, *p* < 0.001; Figure 2b). There were significant strain-dependent differences in central time (F(1,23) = 21.46, *p* < 0.0001) and central entries (F(1,23) = 99.32, *p* < 0.0001), with higher levels found in SHR (Figure 2c,d). Repeated testing significantly reduced the distance travelled in the central zone (F(1,23) = 7.16, *p* < 0.02; Figure 2b), central time (F(1,23) = 16.63, *p* < 0.0005; Figure 2c), and central entries (F(1,23) = 43.6, *p* < 0.0001; Figure 2d) in both strains; however, treatment and interaction of the factors were insignificant, and no differences in the above-mentioned parameters were found among the groups. Importantly, USPION-treatment did not alter the above-mentioned variables, compared to the control groups, in the respective rat strains.

### 3.2. Blood Pressure and Heart Rate

**Figure 3:**
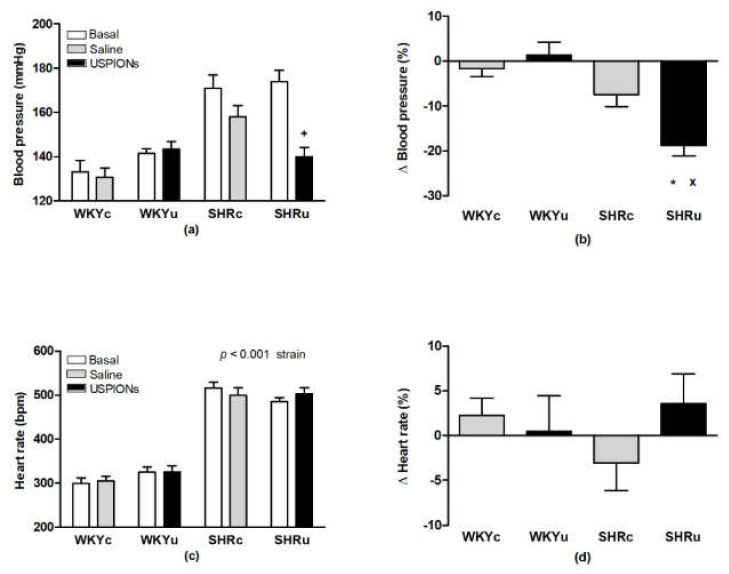
Blood pressure (**a**), relative blood pressure changes (**b**), heart rate (**c**), and relative heart rate (**d**) in rats. Results are mean ± SEM. See the Results section for detailed ANOVA results. + *p* < 0.05 vs. basal, * *p* < 0.05 vs. SHRc, ^x^ *p* < 0.05 vs. WKYu. Abbreviations: USPIONs, ultra-small superparamagnetic iron oxide nanoparticles; WKYc, control Wistar–Kyoto rats; WKYu, USPION-treated Wistar–Kyoto rats; SHRc, control spontaneously hypertensive rats; SHRu, USPION-treated spontaneously hypertensive rats.

ANOVA revealed significant strain-dependent differences in BP of rats (F(1,26) = 32.22, *p* < 0.001) with significantly higher BP in SHR (Figure 3a). The main effect of treatment was not statistically significant. There was a significant effect of repeated BP measurement (F(1,26) = 28.71, *p* < 0.001). ANOVA also found a significant effect of interaction of strain, measurement, and treatment (F(1,26) = 7.29, *p* < 0.02). The BP of USPION-treated SHR was significantly reduced, compared to their basal levels, which was not observed in the SHRc (Figure 3a). Calculation of the BP changes relative to their basal BP levels (Δ BP in percentage of basal levels) showed a significant decrease in BP in the SHRu group vs. both WKYu and SHRc groups (Figure 3b). The HR of SHR was also significantly higher than in WKY (F(1,26) = 265, *p* < 0.0001; main effect of strain). The effects of measurement and treatment, as well as the interactions of all factors, were insignificant (Figure 3c). The relative changes of HR related to the basal levels (Δ HR in percentage of basal levels) did not differ among the groups (Figure 3d).

### 3.3. NO Synthase Activity

**Figure 4:**
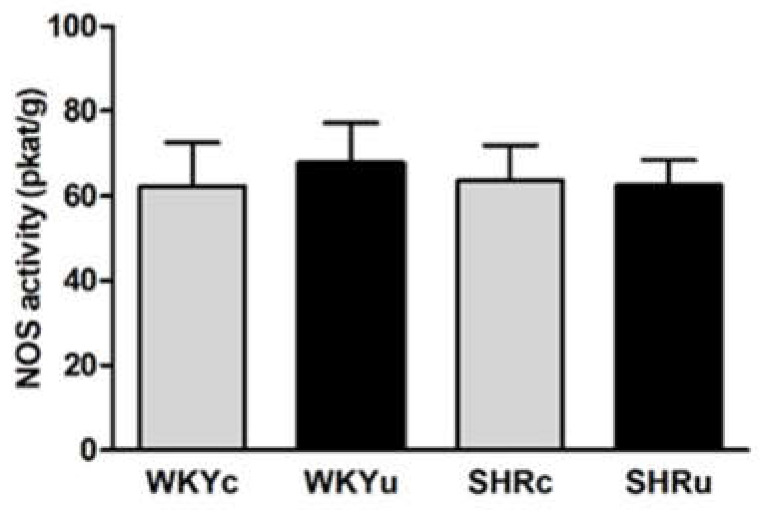
Nitric oxide synthase activity in the brainstem. Results are mean ± SEM. Abbreviations: NOS, nitric oxide synthase activity; USPIONs, ultra-small superparamagnetic iron oxide nanoparticles; WKYc, control Wistar–Kyoto rats; WKYu, USPION-treated Wistar–Kyoto rats; SHRc, control spontaneously hypertensive rats; SHRu, USPION-treated spontaneously hypertensive rats.

**Figure 5:**
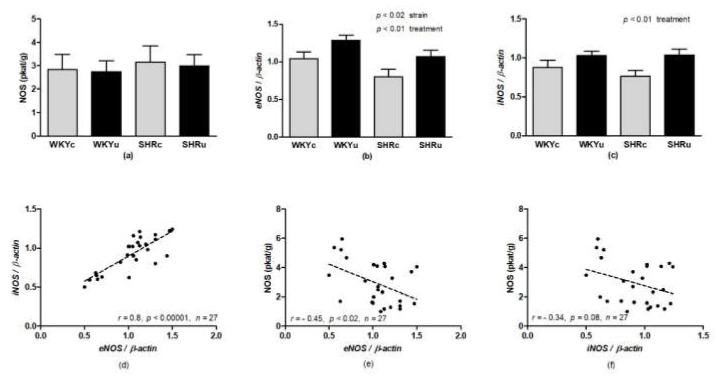
Nitric oxide synthase activity in the aorta (**a**), *eNOS* gene expression (**b**) *iNOS* gene expression (**c**), correlation between *eNOS* and *iNOS* gene expression (**d**), correlation between *eNOS* gene expression and total NOS activity (**e**), and correlation between *iNOS* gene expression and total NOS activity (**f**). Results are mean ± SEM. See the Results section for detailed 2-way ANOVA results. Abbreviations: *eNOS*, endothelial nitric oxide synthase; *iNOS*, inducible nitric oxide synthase; NOS, nitric oxide synthase; USPIONs, ultra-small superparamagnetic iron oxide nanoparticles; WKYc, control Wistar–Kyoto rats; WKYu, USPION-treated Wistar–Kyoto rats; SHRc, control spontaneously hypertensive rats; SHRu, USPION-treated spontaneously hypertensive rats.

**Figure 6:**
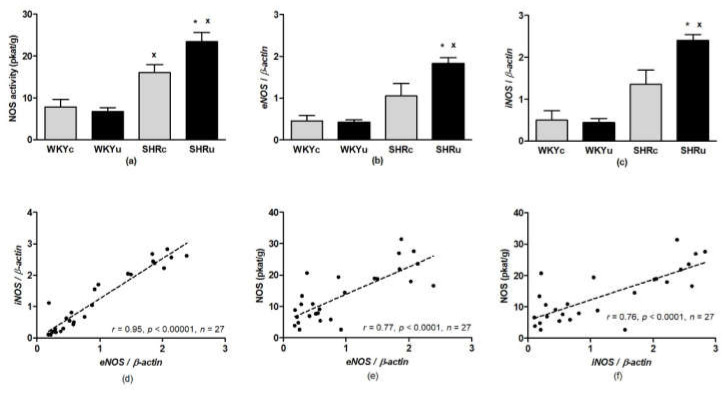
Nitric oxide synthase activity in the liver (**a**), *eNOS* gene expression (**b**) *iNOS* gene expression (**c**), correlation between *eNOS* and *iNOS* gene expression (**d**), correlation between *eNOS* gene expression and total NOS activity (**e**), and correlation between *iNOS* gene expression and total NOS activity (**f**). Results are mean ± SEM. See the Results section for detailed 2-way ANOVA results. * *p* < 0.05 vs. SHRc, ^x^ *p* < 0.05 vs. the respective WKY group. Abbreviations: *eNOS*, endothelial nitric oxide synthase; *iNOS*, inducible nitric oxide synthase; NOS, nitric oxide synthase; USPIONs, ultra-small superparamagnetic iron oxide nanoparticles; WKYc, control Wistar–Kyoto rats; WKYu, USPION-treated Wistar–Kyoto rats; SHRc, control spontaneously hypertensive rats; SHRu, USPION-treated spontaneously hypertensive rats.

NOS activity was determined in the brainstem (due to its function in cardiovascular and motor control), liver (due to its function in the excretion/metabolism of USPIONs), and aorta (due to its function in cardiovascular regulation). ANOVA did not reveal significant differences in NOS activity in the brainstem (Figure 4) and aorta (Figure 5a). In the liver, ANOVA revealed a significant effect of strain (F(1,24) = 50.64, *p* < 0.0001), with higher levels found in SHR. In addition, there was significant effect of interaction of strain and treatment (F(1,24) = 5.79, *p* < 0.03) with NOS activity, which was significantly elevated in SHRu vs. both SHRc and WKYu (Figure 6a).

### 3.4. Gene Expression

Two-way ANOVA revealed a significant effect of strain for *eNOS* gene expression in the aorta, with reduced mRNA levels found in SHR vs. WKY (F(1,23) = 6.73, *p* < 0.02; Figure 5b). In addition, *eNOS* and *iNOS* expressions were elevated in the aorta of USPION-treated rats (*eNOS*: F(1,23) = 8.31, *p* < 0.01; *iNOS*: F(1,23) = 7.76, *p* < 0.02; Figure 5b,c). There was a significant positive correlation between *eNOS* and *iNOS* expression (Figure 5d); however, *eNOS* expression correlated negatively with total NOS activity (Figure 5e) and a trend of negative correlation was found between *iNOS* and total NOS activity in the aorta (Figure 5f).

In the liver, 2-way ANOVA revealed the significant effect of strain and treatment interactions for both *eNOS* (*eNOS*: F(1,23) = 4.95, *p* < 0.04; Figure 6b) and *iNOS* (*iNOS*: F(1,23) = 6.19, *p* < 0.03; Figure 6c). In the SHRu group, gene expressions of *eNOS* and *iNOS* were elevated significantly, compared to both SHRc and WKYu (*p* < 0.04 for all comparisons). There was a significant positive correlation between *eNOS* and *iNOS* expressions (Figure 6d). In contrast to the aorta, *eNOS* and *iNOS* gene expressions correlated positively with total NOS activity (Figure 6e,f).

**Figure 7:**
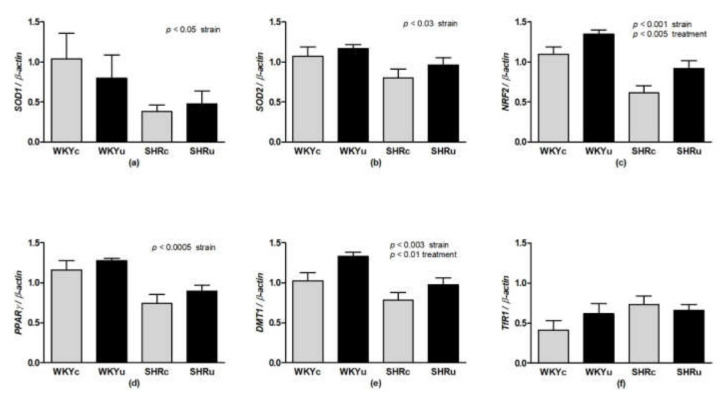
Gene expressions of *SOD1* (**a**), *SOD2* (**b**), *NRF2* (**c**), *PPARγ* (**d**), *DMT1* (**e**), and *TfR1* (**f**) genes in the aorta. Results are mean ± SEM. See the Results section for detailed 2-way ANOVA results. Abbreviations: *NRF2*, nuclear factor erythroid 2–related factor 2; *PPARγ*, peroxisome proliferator-activated receptor gamma; *SOD1*, superoxide dismutase 1; *SOD2*, superoxide dismutase 2; *DMT1*, divalent metal transporter 1; *TfR1*, transferrin receptor 1; USPIONs, ultra-small superparamagnetic iron oxide nanoparticles; WKYc, control Wistar–Kyoto rats; WKYu, USPION-treated Wistar–Kyoto rats; SHRc, control spontaneously hypertensive rats; SHRu, USPION-treated spontaneously hypertensive rats.

Two-way ANOVA revealed a significant effect of strain for mRNA of antioxidant enzymes *SOD1* (F(1,23) = 4.66, *p* < 0.05; Figure 7a), *SOD2* (F(1,23) = 5.62, *p* < 0.03; Figure 7b), nuclear factors *NRF2* (F(1,23) = 27.48, *p* < 0.001; Figure 7c), *PPARγ* (F(1,23) = 17.97, *p* < 0.001; Figure 7d), and *DMT1* (F(1,23) = 11.33, *p* < 0.003; Figure 7e) in the aorta. The mRNA levels of these were significantly reduced in SHR compared to WKY. The effect of treatment was significant in *DMT1* (F(1,23) = 7.93, *p* < 0.01) and *NRF2* (F(1,23) = 9.98, *p* < 0.005), in which USPIONs elevated the mRNA expression levels of both genes in the aorta. No changes in *TfR1* gene expression were found in the aorta (Figure 7f).

**Figure 8:**
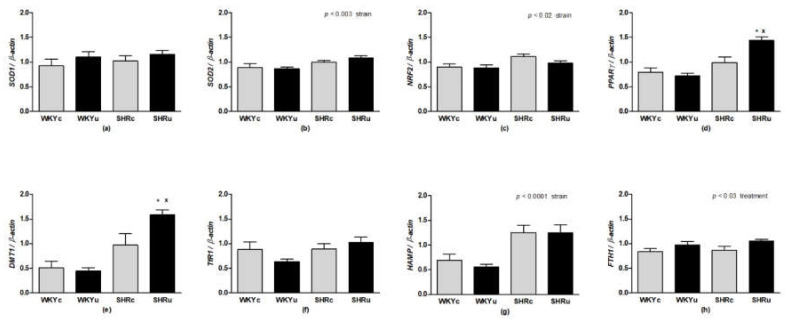
Gene expression of *SOD1* (**a**), *SOD2* (**b**), *NRF2* (**c**), *PPARγ* (**d**), *DMT1* (**e**) *TfR1* (**f**), *HAMP* (**g**), and *FTH1* (**h**) in the liver. Results are mean ± SEM. See the Results section for detailed 2-way ANOVA results. * *p* < 0.05 vs. SHRc, ^x^ *p* < 0.05 vs. WKYu. Abbreviations: *NRF2*, nuclear factor erythroid 2–related factor 2; *PPARγ*, peroxisome proliferator-activated receptor gamma; *SOD1*, superoxide dismutase 1; *SOD2*, superoxide dismutase 2; *DMT1*, divalent metal transporter 1; *TfR1*, transferrin receptor 1; *HAMP*, hepcidin; *FTH1*, ferritin H1 subunit; USPIONs, ultra-small superparamagnetic iron oxide nanoparticles; WKYc, control Wistar–Kyoto rats; WKYu, USPION-treated Wistar–Kyoto rats; SHRc, control spontaneously hypertensive rats; SHRu, USPION-treated spontaneously hypertensive rats.

In the liver, no significant differences in *SOD1* mRNA expression were found (Figure 8a), while a significant effect of strain was found for mRNA expression of *SOD2* (F(1,23) = 11.92, *p* < 0.003; Figure 8b). Strain-dependent effects were found for mRNA expressions of *NRF2* (F(1,23) = 6.84, *p* < 0.02; Figure 8c) and *PPARγ* (F(1,23) = 17.97, *p* < 0.001; Figure 8d), with higher levels in SHR rats. In addition, a significant effect of treatment (F(1,23) = 4.83.97, *p* < 0.04) and interaction of the strain and treatment (F(1,23) = 9.45, *p* < 0.006) was found for the mRNA expression of *PPARγ* in the liver. The effects of strain (F(1,23) = 28.75, *p* < 0.0001; Figure 8e) as well as strain and treatment interaction, was revealed for *DMT1* expression (F(1,23) = 5.15, *p* < 0.04; Figure 8e). Similarly to the aorta, no changes in *TfR1* were found among the groups (Figure 8f). Strain-dependent effects was found also for *HAMP* expression (F(1,23) = 22.98, *p* < 0.0001; Figure 8g). Treatment had a significant effect on *FTH1* mRNA expression (F(1,23) = 6.05, *p* < 0.03; Figure 8h), where higher levels were found in USPION-treated rats.

### 3.5. Vascular Function

Two-way ANOVA showed significant strain-dependent differences in KPSS-induced contractions (F(1,44) = 80.14, *p* < 0.0001), with higher levels in SHR compared to WKY (Table 3).

**Figure 9:**
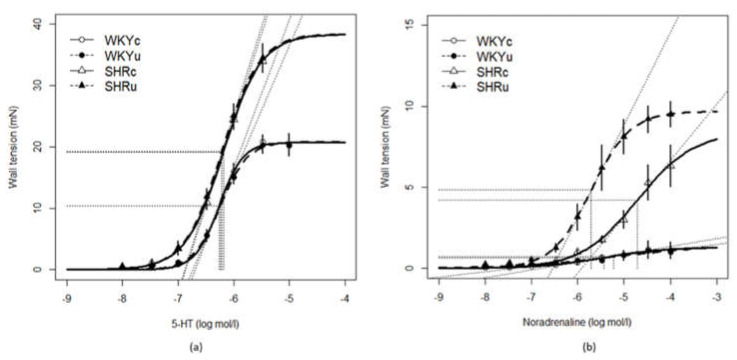
5-hydroxytryptamine—(**a**) and noradrenaline-induced (**b**) contractions in the femoral arteries in the control and USPION-treated rats. Abbreviations: WKYc, control Wistar–Kyoto rats; WKYu, USPION-treated Wistar–Kyoto rats; SHRc, control spontaneously hypertensive rats; SHRu, USPION-treated spontaneously hypertensive rats; NA, noradrenaline; 5-HT, 5-hydroxytryptamine (serotonin).

The EC_50_ and Emax of 5-HT-induced contraction were elevated in SHR, compared to WKY (EC_50_: F(1,23) = 4.54, *p* < 0.05; E_max_: F(1,23) = 65.28, *p* < 0.0001). No significant effects of treatment or interaction of the strain and treatment were found for these parameters (Table 3, Figure 9a). In contrast, there were significant differences in the interaction between the strain and treatment for EC_50_, E_max_, and slope of NA-induced contractions (EC_50_: F(1,21) = 6.56, *p* < 0.02; E_max_: F(1,21) = 4.40, *p* < 0.05, slope: F(1,21) = 8.03, *p* < 0.01; Table 3). In addition, there were significant strain-dependent differences in the E_max_ and slope of NA-induced contractions (E_max_: F(1,21) = 55.44, *p* < 0.0001, slope: F(1,21) = 26.50, *p* < 0.0001), as well as treatment-dependent differences (E_max_: F(1,21) = 7.38, *p* < 0.03; slope: F(1,21) = 7.13, *p* < 0.02; Table 3, Figure 9b).

**Figure 10:**
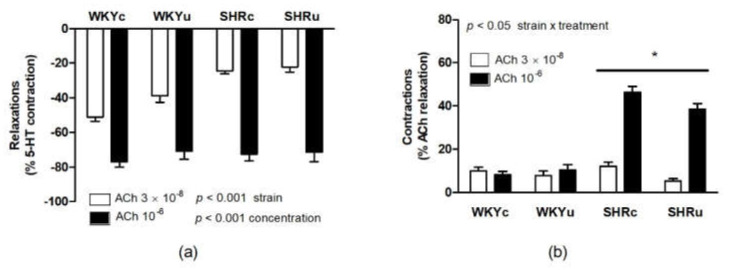
Acetylcholine-induced endothelium-dependent relaxation (**a**) and acetylcholine-induced endothelium-dependent contractions (**b**) in the femoral arteries. The values represent the mean ± SEM. See the Results section for detailed 2-way ANOVA results. * *p* < 0.02 SHRc vs. SHRu. Abbreviations: ACh, acetylcholine; 5-HT, 5-hydroxytryptamine; USPIONs, ultra-small superparamagnetic iron oxide nanoparticles; WKYc, control Wistar–Kyoto rats; WKYu, USPION-treated Wistar–Kyoto rats; SHRc, control spontaneously hypertensive rats; SHRu, USPION-treated spontaneously hypertensive rats.

ACh-induced relaxations were investigated in the arterial segments after 5-HT-induced contraction. There were obvious strain-dependent differences in ACh-induced relaxations, which were reduced in SHR vs. WKY (F(1,23) = 14.58, *p* < 0.001; main effect of strain, Figure 10a). Higher ACh concentration resulted in higher relaxations (F(1,21) = 389.74, *p* < 0.0001; main effect of concentration) in both rat strains (Figure 10a).

As high ACh concentration is known to induce the release of endothelium-derived contracting factors [21,28], endothelium-dependent contractions of the arterial segments induced by high ACh dose were investigated (Figure 10b). There was a significant effect of strain (F(1,23) = 93.03, *p* < 0.0001; with higher contractions in SHR vs. WKY), and the effect of treatment (F(1,23) = 4.37, *p* < 0.05) with lower contractions in USPION-treated rats vs. controls. The effect of the interaction between strain and treatment was also significant (F(1,23) = 4.52, *p* < 0.05), and USPIONs reduced the endothelium-dependent contractions after both applications of ACh in SHRu vs. SHRc (*p* < 0.02), while no differences were found in WKY (Figure 10b).

### 3.6. USPION-Originated Iron Content

**Figure 11:**
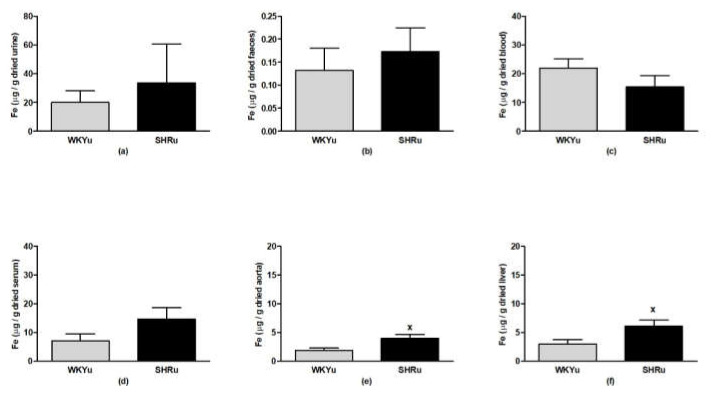
USPION-originated iron content in urine (**a**), faeces (**b**), blood (**c**), serum (**d**), aorta (**e**), and liver (**f**). The values represent the mean ± SEM. ^x^ *p* < 0.05 vs. WKYu, *n* = 5–6/group. Abbreviations: WKYu, USPION-treated Wistar–Kyoto rats; SHRu, USPION-treated spontaneously hypertensive rats.

In urine, faeces, blood, and serum, USPION-originated iron content did not differ significantly between WKYu and SHRu rats (Figure 11a–d). USPION-originated iron content was significantly elevated in the aorta (*p* < 0.05; Figure 11e) and liver (*p* < 0.04; Figure 11f) of SHRu rats vs. WKYu, by 109% and 101%, respectively.

### 3.7. Electron Microscopy

**Figure 12:**
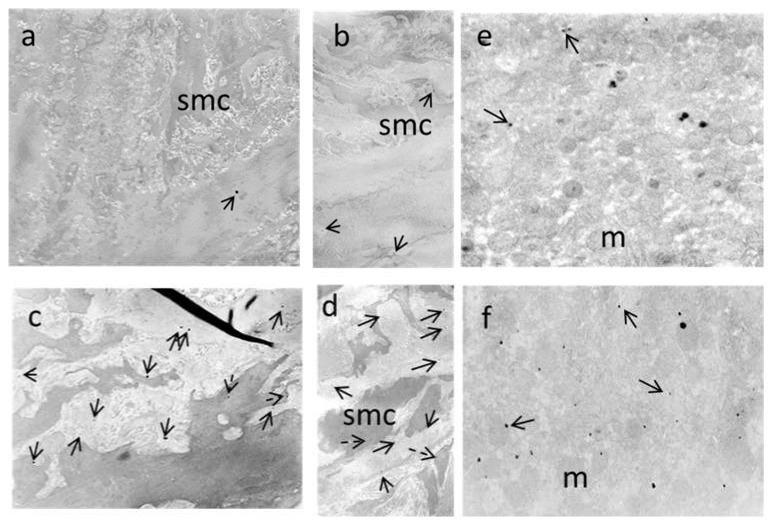
Representative electron micrographs of the unstained ultra-thin sections of the aorta of USPION-treated Wistar–Kyoto rats (**a**,**b**) demonstrating distribution of nanoparticles (solid arrows) within the elastic layers. In USPION-treated spontaneously hypertensive rats, nanoparticles were present in the smooth muscle cells (dashed arrows), as well as within the elastic layers (solid arrows) (**c**,**d**). In the liver, a heterogeneous distribution of nanoparticles was observed in hepatocytes of WKY (**e**) and SHR (**f**). Original magnifications: ×4000. Abbreviations: smc, vascular smooth muscle cells; m, mitochondria.

A semi-quantitative analysis of USPION content in the aorta and liver demonstrated a higher number of nanoparticles in both tissues of SHRu than in WKYu (Table 4). Electron microscopy revealed heterogeneous distributions of USPIONs in the aortic wall and liver of both strains. In WKY rats, USPIONs were observed in the elastic layers of the aorta (Figure 12a,b), while in the aorta of SHR, the USPIONs were localized in both the elastic layers and smooth muscle cells (Figure 12c,d). In the liver, USPIONs were present in the hepatocytes of both strains (Figure 9e,f).

## 4. Discussion

In this work, we evaluated the tissue distribution and selected biological effects of Fe_3_O_4_@PEG nanoparticles in normotensive WKY and spontaneously hypertensive rats. The main findings of our study were as follows: (1) USPIONs did not alter the spontaneous open-field behaviour in both rat strains; (2) hypertension was associated with increased deposition of USPIONs into the aortic vascular wall and liver; and (3) USPIONs elevated NA-induced contractions in isolated femoral artery, but reduced endothelium-dependent contractions and blood pressure in hypertensive rats. In addition, we found elevated NOS activity in the liver of SHRu, associated with elevated gene expression of *eNOS*, *iNOS*, and *DMT1* in this organ, while no changes in NOS activities were found in the aorta and brainstem of any rat strain.

As first, we evaluated the possible USPION-induced behavioural changes in an open-field test. We found no changes in the parameters associated with locomotor activity and anxiety-like behaviour in WKY. Similarly, Askri et al. [29] have shown that sub-acute intranasal exposure to IONs did not affect the emotional behaviour, anxiety index, and learning/memory capacities of Wistar rats. In addition, low-dose IONs failed to alter behaviour in open-field and forced swim tests in Wistar rats [30]. On the other hand, high doses of uncoated IONs administered to mice i.p. once a week for 4 weeks led to damage to the blood–brain barrier, altering locomotor behaviour and spatial memory [31]. To our knowledge, this study is the first dealing with the behavioural effects of Fe_3_O_4_@PEG in SHR. Despite significant locomotor hyperactivity and damage to the blood–brain barrier, both of which are well-known in SHR [32,33,34], repeated i.v. administration of USPIONs did not alter the open-field behaviour of rats in terms of locomotion or parameters associated with anxiety-like behaviour. In addition, no changes in NO production were found in the brainstem, which was selected due to its roles in regulating both motoric functions and BP.

Regarding BP, USPIONs significantly reduced BP in SHR but not in WKY. In our previous studies, we found a delayed decrease in BP in acute stress-exposed USPION-treated WKY rats, but not in rats treated with USPIONs under control conditions [19]. Using polyacrylic acid-coated γ-Fe_2_O_3_ NPs, Iversen et al. have reported a transient decrease in BP when determined 12–24 h after administration in mice [35]. In human studies, hypotension has been observed after administration of IONs to improve magnetic resonance imaging contrast [21]. On the other hand, acute intravenous administration of Fe_3_O_4_@PEG-Alendronate NPs to SHR rats did not produce changes in BP when investigated 100 min after acute i.v. infusion [36]. In this study, a decrease in BP occurred only in SHR 24 h post-treatment, suggesting possible changes in vascular functions.

For the investigation of vascular function, we used the femoral artery, the tone of which is regulated by both the endothelium-derived relaxing and contracting factors [19], making it ideal for the study of vascular responses in various experimental approaches. Previously, we found that an acutely infused lower dose of the USPIONs did not alter 5-HT-induced contractions and overall endothelium-dependent ACh-induced relaxations but elevated its NO-dependent component in WKY. Similarly, Fe_3_O_4_@PEG-alendronate-coated NPs did not damage the vascular function of the femoral artery in SHR after a single infusion [36]. In this study, 5-HT-induced contractions were unaltered by repeated USPION administration, while NA-induced contractions were significantly increased, compared to the control SHR, suggesting elevated sympathetic nervous system tone. On the other hand, ACh-induced endothelium-dependent contractions were reduced in USPION-treated SHR, possibly indicating a compensatory mechanism. Regarding USPION localisation, we showed that USPIONs were present only in a smaller number, and dominantly in the elastic layers, in USPION-treated WKY rats. However, in SHR, USPIONs were present in a greater amount in both the elastic layers and vascular smooth muscle cells. The elevated amount of USPION-originated iron in the aorta of SHRu, compared to WKYu was also confirmed using a magnetometric method. This may result from hypertension, which can alter or damage cell-to-cell connections [37], thus allowing for increased incorporation of USPIONs into the vascular wall. In addition, the permeability of vascular smooth muscle cell membranes may also be increased under hypertensive conditions, which may allow USPIONs to enter the vascular smooth muscle cells. In agreement with these findings, we also found elevated gene expression of *DMT1* (involved in the transport of divalent metals, including Fe^2+^), as well as nuclear factors *NRF2* and *PPARγ*—which are involved in the regulation of various genes, including those involved in antioxidant defence and iron metabolism [14,38,39]—when calculated as the main effect of treatment, in both rat strains. Such changes were associated with elevated intracellular levels of iron. We also observed a significant main effect of USPIONs on *eNOS* and *iNOS* gene expression in the aorta, both of which were negatively correlated with total NOS activity (which remained unchanged). As the expression of *SOD1* and *SOD2* were also unchanged, USPIONs supposedly did not induce elevated superoxide production, which could react with NO. Thus, NO bioavailability should remain unchanged, which was supported by the finding of unchanged ACh-induced relaxation in the femoral artery. Collectively, despite higher incorporation of USPIONs into the vascular wall and smooth muscle cells in SHR, there were no changes in NO bioavailability in the aorta and ACh-induced relaxations in the femoral artery. Thus, the above-mentioned alterations in the arteries cannot explain the BP decrease in USPION-treated SHR, without the contribution of other factors.

Such an important factor could be alterations in the liver vascular bed and renal and hepatic function. The liver is well-known as the main organ for excretion of USPIONs of the size used in this study. However, the decomposition of USPIONs can result in renal excretion, which we observed in both rat strains. This, however, was not associated with significant kidney damage as we have published previously [26]. Regarding the liver, we found that NO synthase activity was significantly elevated in the USPION-treated SHR, which was correlated positively with elevated *iNOS* and *eNOS* gene expression. Thus, a factor considerably contributing to the decrease in BP in USPION-treated rats may be elevated relaxation in the hepatic vasculature resulting from elevated NO production by both eNOS and iNOS [23], further reducing peripheral vascular resistance. In addition, elevated expression of *PPARγ* may suggest early stages of liver damage [17,18].

We also found that USPIONs increased the gene expression of *DMT1* in SHRu and elevated gene expression of *FTH1* (main effect of treatment) in the liver, which may suggest elevated iron levels in this organ. Interestingly, the gene expression of *HAMP* gene, encoding hepcidin, a main hormone produced in the liver that regulates iron absorption in enterocytes and other tissues, was unaltered in both rat strains. On the basis of these findings, we can assume that USPIONs did not modify the signal pathways involved in hepcidin release in the liver at this stage of their action, as iron from (at least partially) decomposed USPIONs was successfully trapped in ferritin cores and/or excreted. The unchanged hepcidin levels were also in agreement with our finding of unaltered blood content of USPIONs in SHRu. Importantly, our previous studies showed a reduced amount of USPION-originated iron in erythrocytes in SHRu compared to WKYu [26]. This is in agreement with the trend of elevated USPION levels in sera of SHRu in this study, suggesting differences in erythrocyte membrane properties resulting in higher binding of USPIONs on erythrocytes of WKY rats, compared to SHR. Higher amount of USPIONs unbound to erythrocytes can, then, lead to elevated USPION incorporation into the tissues. The higher amount of USPIONs in the bloodstream of SHR may also lead to partially elevated renal and hepatic excretion of USPIONs in SHRu. However, the absolute amounts of USPION-originated iron in renal and hepatic excretions from both rat strains remain unknown, as we did not collect all urine and faeces during the experiment for calculation of these parameters.

## 5. Conclusions

In conclusion, our results showed an altered distribution of USPIONs in SHRu, compared to WKYu; namely, elevated USPION incorporation into the vascular wall and the liver, supposedly as a result of a higher amount of erythrocyte-unbound NPs in the bloodstream of SHR. This could lead to increase in NO production in the liver, due to the activation of *iNOS* and *eNOS* gene expression, resulting in lower peripheral vascular resistance associated with BP decrease only in SHR. Thus, our findings suggest caution when using Fe_3_O_4_@PEG-coated NPs in hypertensive subjects, as they may produce a BP decrease.

## Figures and Tables

**Table 1:** Experimental protocol.

Day	Procedure
−3	07:00–07:30 h Open-field test (basal)
−2	08:30–09:00 h Blood pressure determination (basal)
−1	13:00–15:00 h Catheterization
0	07:00 h Infusion—the first USPION (or saline) dose
1	07:00 h Infusion—the second USPION (or saline) dose
2	07:00 h Open-field test (end) 09:00 h Blood pressure determination (end) 09:30 h Decapitation

**Table 2:** Primer pairs used to amplify selected genes.

Gene	Forward Primer	Reverse Primer	Tm (°C)	Amp (bp)
*iNOS* (NM_012611.3)	AAA CGC TAC ACT TCC AAC GC	TGC TGA GAG CTT TGT TGA GGT C	59	91
*eNOS* (NM_021838.2)	GAT CCC CCG GAG AAT GGA GA	TCG GAT TTT GTA ACT CTT GTG CT	60	105
*NRF2* (NM_021838.2)	TGC CAT TAG TCA GTC GCT CTC	ACC GTG CCT TCA GTG TGC	60	102
*SOD1* (NM_017050.1)	CTG AAG GCG AGC ATG GGT TC	TCC AAC ATG CCT CTC TTC ATC C	60	131
*SOD2* (NM_017051.2)	GCT GGC CAA GGG AGA TGT TAC	TGC TGT GAT TGA TAT GGC CCC	60	83
*PPARγ* (NM_013124.3)	CTC ACA ATG CCA TCA GG TTT GG	GCT GGT CGA TAT CAC TGG AGA T	59	84
*DMT1* (NM_013173.2)	CTA CTT GGG TTG GCA GTG TTT G	ATC TTC GCT CAG CAG GAC TTT	60	94
*TFR1* (NM_022712.1)	GCT ATG AGG AAC CAG ACC GC	CAC TGG ACT TCG CAA CAC CA	58	78
*β-actin* (NM_031144.3)	CTC TGT GTG GAT TGG TGG CT	CGC AGC TCA GTA ACA GTC CG	59	139

**Table 3:** Parameters of depolarising solution-, 5-hydroxytryptamine-, and noradrenaline-induced contractions in the femoral arteries.

5-HT				
	WKYc *n* = 6	WKYu *n* = 7	SHRc *n* = 7	SHRu *n* = 7
KPSS (mN/mm) ^+^	14.15 ± 0.89	13.88 ± 0.90	28.21 ± 1.94	26.95 ± 2.88
E_max_ (%) ^+^	20.78 ± 1.21	20.12 ± 1.53	36.26 ± 2.11	38.44 ± 2.86
EC_50_ (log mol/l) ^+^	−6.25 ± 0.05	−6.24 ± 0.04	−6.16 ± 0.05	−6.17 ± 0.03
slope	25.07 ± 2.62	22.89 ± 1.47	32.42 ± 2.51	30.24 ± 2.31
NA				
	WKYc *n* = 6	WKYu *n* = 6	SHRc *n* = 6	SHRu *n* = 7
KPSS (mN/mm) ^+^	19.08 ± 0.84	17.34 ± 0.79	26.84 ± 1.73	25.15 ± 1.63
E_max_ (%)	1.38 ± 0.65	1.93 ± 0.36	6. 12 ± 1.35 ^x^	10. 39 ± 0.86 ^x,^*
EC_50_ (log mol/l)	−5.66 ± 0.41	−5.10 ± 0.34	−4.63 ± 0.13 ^x^	−5.55 ± 0.34 *
slope	0.60 ± 0.28	0.44 ± 0.09	2.89 ± 0.50	8.33 ± 1.67 *

Values represent the mean ± SEM. * *p* < 0.05 vs. the SHRc, ^x^ *p* < 0.05 vs. the respective WKY group. A “^+^” means that the main ANOVA effect for the strain is significant, see the Results section for detailed 2-way ANOVA results. Abbreviations: EC_50_, half-maximal effective concentration; E_max_, maximal contraction; KPSS, 125 mmol/L K^+^-containing physiological salt solution (see Methods section for details); NA, noradrenaline; 5-HT, 5-hydroxytryptamine (serotonin); USPIONs, ultra-small superparamagnetic iron oxide nanoparticles; WKYc, control Wistar–Kyoto rats; WKYu, USPION-treated Wistar–Kyoto rats; SHRc, control spontaneously hypertensive rats; SHRu, USPION-treated spontaneously hypertensive rats.

**Table 4:** Semi-quantitative evaluation of the USPIONs in the aorta.

Groups	Aorta			Liver
	Total	Smooth Muscle Cells	Elastic Layers	
WKYu	+	−	+	+, ++
SHRu	+++	+	+++	++,+++

The score was defined as minus (−)—indicating the absence of USPIONs, (+)—indicating weak presence of USPIONs, (++)—indicating moderate presence of USPIONs and, (+++)—indicating the highest amount of USPIONs in the given tissue. Abbreviations: WKYu, USPION-treated Wistar–Kyoto rats; SHRu, USPION-treated spontaneously hypertensive rats.

## Data Availability

The data presented in this study are available on request from the corresponding author.

## References

[1] Andrade R.G.D., Veloso S.R.S., Castanheira E.M.S. (2020). Shape Anisotropic Iron Oxide-Based Magnetic Nanoparticles: Synthesis and Biomedical Applications. Int. J. Mol. Sci..

[2] Poller W.C., Pieber M., Boehm-Sturm P., Ramberger E., Karampelas V., Möller K., Schleicher M., Wiekhorst F., Löwa N., Wagner S. (2018). Very small superparamagnetic iron oxide nanoparticles: Long-term fate and metabolic processing in atherosclerotic mice. Nanomedicine.

[3] Rego G.N.A., Nucci M.P., Mamani J.B., Oliveira F.A., Marti L.C., Filgueiras I.S., Ferreira J.M., Real C.C., Faria D.D.P., Espinha P.L. (2020). Therapeutic Efficiency of Multiple Applications of Magnetic Hyperthermia Technique in Glioblastoma Using Aminosilane Coated Iron Oxide Nanoparticles: In Vitro and In Vivo Study. Int. J. Mol. Sci..

[4] Roca A.G., Marco J.F., Morales M.D.P., Serna C.J. (2007). Effect of Nature and Particle Size on Properties of Uniform Magnetite and Maghemite Nanoparticles. J. Phys. Chem. C.

[5] Larsen E.K.U., Nielsen T., Wittenborn T., Rydtoft L.M., Lokanathan A.R., Hansen L., Østergaard L., Kingshott P., Howard K.A., Besenbacher F. (2012). Accumulation of magnetic iron oxide nanoparticles coated with variably sized polyethylene glycol in murine tumors. Nanoscale.

[6] Suk J.S., Xu Q., Kim N., Hanes J., Ensign L.M. (2016). PEGylation as a strategy for improving nanoparticle-based drug and gene delivery. Adv. Drug Deliv. Rev..

[7] Lee W.-K., Park J.-Y., Jung S., Yang C.W., Kim W.-U., Kim H.-Y., Park J.-H. (2005). Preparation and characterization of biodegradable nanoparticles entrapping immunodominant peptide conjugated with PEG for oral tolerance induction. J. Control. Release.

[8] Malhotra N., Lee J.-S., Liman R.A.D., Ruallo J.M.S., Villaflores O.B., Ger T.-R., Hsiao C.-D. (2020). Potential Toxicity of Iron Oxide Magnetic Nanoparticles: A Review. Molecules.

[9] Drakesmith H., Nemeth E., Ganz T. (2015). Ironing out Ferroportin. Cell Metab..

[10] Wang J., Pantopoulos K. (2011). Regulation of cellular iron metabolism. Biochem. J..

[11] Cuadrado A., Manda G., Hassan A., Alcaraz M.J., Barbas C., Daiber A., Ghezzi P., León R., López M.G., Oliva B. (2018). Transcription Factor NRF2 as a Therapeutic Target for Chronic Diseases: A Systems Medicine Approach. Pharmacol. Rev..

[12] Robledinos-Antón N., Fernández-Ginés R., Manda G., Cuadrado A. (2019). Activators and Inhibitors of NRF2: A Review of Their Potential for Clinical Development. Oxidative Med. Cell. Longev..

[13] Zhou S., Sun W., Zhang Z., Zheng Y. (2014). The Role of Nrf2-Mediated Pathway in Cardiac Remodeling and Heart Failure. Oxidative Med. Cell. Longev..

[14] Kerins M.J., Ooi A. (2018). The Roles of NRF2 in Modulating Cellular Iron Homeostasis. Antioxid. Redox Signal..

[15] Lim P.J., Duarte T.L., Arezes J., Garcia-Santos D., Hamdi A., Pasricha S.-R., Armitage A.E., Mehta H., Wideman S.K., Santos A.G. (2019). Nrf2 controls iron homoeostasis in haemochromatosis and thalassaemia via Bmp6 and hepcidin. Nat. Metab..

[16] Lee C. (2017). Collaborative Power of Nrf2 and PPARγ Activators against Metabolic and Drug-Induced Oxidative Injury. Oxidative Med. Cell. Longev..

[17] Videla L.A., Pettinelli P. (2012). Misregulation of PPAR Functioning and Its Pathogenic Consequences Associated with Nonalcoholic Fatty Liver Disease in Human Obesity. PPAR Res..

[18] Tailleux A., Wouters K., Staels B. (2012). Roles of PPARs in NAFLD: Potential therapeutic targets. Biochim. Biophys. Acta-Mol. Cell Biol. Lipids.

[19] Líšková S., Bališ P., Mičurová A., Kluknavský M., Okuliarová M., Puzserová A., Škrátek M., Sekaj I., Maňka J., Valovič P. (2020). Effect of iron oxide nanoparticles on vascular function and nitric oxide production in acute stress-exposed rats. Physiol. Res..

[20] Skrátek M., Dvurečenskij A., Kluknavský M., Barta A., Balis P., Mičurová A., Cigáň A., Andicsová A.E., Maňka J., Bernátová I. (2020). Sensitive SQUID Bio-Magnetometry for Determination and Differentiation of Biogenic Iron and Iron Oxide Nanoparticles in the Biological Samples. Nanomaterials.

[21] Bernatova I. (2014). Endothelial Dysfunction in Experimental Models of Arterial Hypertension: Cause or Consequence?. BioMed Res. Int..

[22] Puzserova A., Bernatova I. (2016). Blood Pressure Regulation in Stress: Focus on Nitric Oxide-Dependent Mechanisms. Physiol. Res..

[23] El Hadi H., Di Vincenzo A., Vettor R., Rossato M. (2020). Relationship between Heart Disease and Liver Disease: A Two-Way Street. Cells.

[24] Kluknavsky M., Balis P., Puzserova A., Radosinska J., Berenyiova A., Drobna M., Lukac S., Muchova J., Bernatova I. (2016). (−)-Epicatechin Prevents Blood Pressure Increase and Reduces Locomotor Hyperactivity in Young Spontaneously Hypertensive Rats. Oxidative Med. Cell. Longev..

[25] Puzserova A., Slezak P., Balis P., Bernatova I. (2012). Long-term social stress induces nitric oxide-independent endothelial dysfunction in normotensive rats. Stress.

[26] Radosinska J., Jasenovec T., Radosinska D., Balis P., Puzserova A., Skratek M., Manka J., Bernatova I. (2021). Ultra-Small Superparamagnetic Iron-Oxide Nanoparticles Exert Different Effects on Erythrocytes in Normotensive and Hypertensive Rats. Biomedicines.

[27] Okruhlicová L., Cicaková Z., Frimmel K., Weismann P., Krizak J., Sotniková R., Knezl V., Slezák J. (2018). Lipopolysaccharide-induced redistribution of myocardial connexin43 is associated with increased macrophage infiltration in both normotensive and spontaneously hypertensive rats. J. Physiol. Pharm..

[28] Silvia L., Miriam P., Petr K., Jaroslav K., Josef Z. (2011). Effects of aging and hypertension on the participation of endothelium-derived constricting factor (EDCF) in norepinephrine-induced contraction of rat femoral artery. Eur. J. Pharm..

[29] Askri D., Ouni S., Galai S., Arnaud J., Chovelon B., Lehmann S.G., Sturm N., Sakly M., Sève M., Amara S. (2018). Intranasal instillation of iron oxide nanoparticles induces inflammation and perturbation of trace elements and neurotransmitters, but not behavioral impairment in rats. Environ. Sci. Pollut. Res..

[30] Saeidienik F., Shahraki M.R., Fanaei H., Badini F. (2018). The Effects of Iron Oxide Nanoparticles Administration on Depression Symptoms Induced by LPS in Male Wistar Rats. Basic Clin. Neurosci. J..

[31] Dhakshinamoorthy V., Manickam V., Perumal E. (2017). Neurobehavioural Toxicity of Iron Oxide Nanoparticles in Mice. Neurotox. Res..

[32] Sagvolden T., Johansen E.B., Stanford C., Tannock R. (2011). Rat models of ADHD. Behavioral Neuroscience of Attention Deficit Hyperactivity Disorder and Its Treatment. Current Topics in Behavioral Neurosciences.

[33] Pires P., Ramos C.M.D., Matin N., Dorrance A.M. (2013). The effects of hypertension on the cerebral circulation. Am. J. Physiol. Circ. Physiol..

[34] Mayhan W.G., Faraci F., Heistad D.D. (1986). Disruption of the blood-brain barrier in cerebrum and brain stem during acute hypertension. Am. J. Physiol. Circ. Physiol..

[35] Iversen N.K., Frische S., Thomsen K., Laustsen C., Pedersen M., Hansen P.B.L., Bie P., Fresnais J., Berret J.-F., Baatrup E. (2013). Superparamagnetic iron oxide polyacrylic acid coated γ-Fe_2_O_3_ nanoparticles do not affect kidney function but cause acute effect on the cardiovascular function in healthy mice. Toxicol. Appl. Pharm..

[36] Oleksa V., Bernátová I., Patsula V., Líšková S., Bališ P., Radošinská J., Mičurová A., Kluknavský M., Jasenovec T., Radošinská D. (2021). Poly(ethylene glycol)-Alendronate-Coated Magnetite Nanoparticles Do Not Alter Cardiovascular Functions and Red Blood Cells’ Properties in Hypertensive Rats. Nanomaterials.

[37] Dlugosova K., Okruhlicova L., Mitasikova M., Sotnikova R., Bernatova I., Weismann P., Slezak J., Tribulova N. (2009). Modulation of connexin-43 by omega-3 fatty acids in the aorta of old spontaneously hypertensive rats. J. Physiol. Pharm..

[38] Kaspar J.W., Niture S.K., Jaiswal A.K. (2009). Nrf2:INrf2 (Keap1) signaling in oxidative stress. Free. Radic. Biol. Med..

[39] Janani C., Kumari B.R. (2015). PPAR gamma gene—A review. Diabetes Metab. Syndr. Clin. Res. Rev..

